# Donepezil modulates amyloid precursor protein endocytosis and reduction by up-regulation of SNX33 expression in primary cortical neurons

**DOI:** 10.1038/s41598-019-47462-4

**Published:** 2019-08-15

**Authors:** Yuki Takada-Takatori, Shota Nakagawa, Riko Kimata, Yousuke Nao, Yumiko Mizukawa, Tetsuro Urushidani, Yasuhiko Izumi, Akinori Akaike, Katsuharu Tsuchida, Toshiaki Kume

**Affiliations:** 1 Faculty of Pharmaceutical Sciences, Doshisha Women’s College, Kyoto, 610-0395 Japan; 2 Department of Pharmacology, Graduate School of Pharmaceutical Sciences, Kyoto University, Kyoto, 606-8501 Japan; 3 Laboratory of Pharmacology, Kobe Pharmaceutical University, Hyogo, 658-8558 Japan; 4 Department of Pharmacology, Graduate School of Medicine, Wakayama Medical University, Wakayama, 641-0012 Japan; 5 Department of Applied Pharmacology, Graduate School of Medical and Pharmaceutical Sciences, University of Toyama, Toyama, 930-0194 Japan

**Keywords:** Neuroscience, Gene expression

## Abstract

Donepezil, a therapeutic drug for Alzheimer’s disease, ameliorates cognitive dysfunction through selective inhibition of acetylcholinesterase. However, recent studies have also reported off-target effects of donepezil that likely contribute to its therapeutic effects. In this study, we investigated the (i) role of donepezil in amyloid precursor protein (APP) processing and (ii) involvement of sorting nexin protein 33 (SNX33), a member of the sorting nexin protein family, in this processing. Results showed that donepezil induces an increase in SNX33 expression in primary cortical neurons. The secretion of sAPPα in culture media increased, whereas the expression of full-length APP in the cell lysate remained unchanged. Exposure of cortical cultures to donepezil led to a decrease in amyloid β (Aβ) protein levels in a concentration- and time-dependent manner. This decrease was not affected by concomitant treatment with acetylcholine receptor antagonists. SNX33 knockdown by target-specific morpholino oligos inhibited the effects of donepezil. Donepezil treatment increased cell membrane surface expression of APP in SNX33 expression-dependent manner. These results suggested that donepezil decreases the level of Aβ by increasing SNX33 expression and APP cleavage by α-secretase in cortical neurons.

## Introduction

Alzheimer’s disease (AD) is a progressive neurodegenerative disorder characterised by depletion of high-affinity nicotinic acetylcholine receptors (nAChRs), marked loss of cholinergic neurons, occurrence of neurofibrillary tangles and accumulation of senile plaques composed of extracellular deposits of amyloid β (Aβ) protein^1–3^.Despite disagreements with regard to the precise nature and role of Aβ (the product of proteolytic cleavage of amyloid precursor protein [APP]) in the brain, the therapeutic goal, in general, has been to reduce Aβ levels, especially under pathological conditions^4^. APP is a type I membrane protein which is subjected to a complex array of proteolytic cleavage, often called ‘ectodomain shedding’, resulting in various soluble counterparts^5^. The amyloidogenic pathway involves two sequential cleavages by β- and γ-secretases and produces Aβprotein. β-secretase cleaves APP at the amino-terminal side of the Aβ sequence, which is within APP, and generates a carboxyl-terminal fragment, C99, which is then cleaved by γ-secretase, resulting in production of Aβprotein. Conversely, the non-amyloidogenic pathway involves α-secretase, which cleaves APP within the Aβsequence. This precludes Aβ generation and generates a soluble secreted form of the APP fragment, sAPPα, which possesses neurotrophic and neuroprotective properties^4^. The two pathways occur in two distinct cellular locations: cleavage by α-secretase occurs at the plasma membrane, whereas β-secretase cleavage occurs mainly in the endosome^5^. It has been believed that the balance between the two cleavage pathways likely determines the production level of the Aβ protein. Several studies on endocytic transport factors have shown that endocytic transport is a critical factor determining the balance between the two pathways^6–9^.

Many aspects of the regulatory mechanism governing APP endocytosis are still unclear. However, we are beginning to understand the factors involved in endocytic trafficking and their roles. The sorting nexin protein (SNX) family comprises endosomal trafficking proteins that share a common phox-homology (PX) domain^10,11^. SNX33, along with SNX9 and SNX18, forms a subfamily characterised by the presence of Src homology 3 (SH3), PX and Bin/amphiphysin/Rvs domains^10^. SNX33 is a recent addition to the subfamily and modifies APP processing. In addition to increasing APP cleavage by α-secretase, SNX33 reduces APP endocytosis and thereby likely decreases shedding by β-secretase^12^. SNX33 also inhibits the function of dynamin, a key component of endocytosis^13^, by directly binding to dynamin through its SH3 domain. Dynamin mutants and dominant negative forms, in turn, increase APP cleavage by α-secretase, reduce APP endocytosis and decrease shedding by β-secretase^7,8,12^. These results suggest that SNX33 modifies processing of APP in the direction of the non-amyloidogenic pathway via the inhibition of APP endocytosis^5,12^.

Donepezil is an acetylcholinesterase (AChE) inhibitor designed for AD treatment. Donepezil moderately improves cognitive function, with relatively low side effects compared with other AD drugs^14,15^. Studies have shown that in addition to being an AChE inhibitor, donepezil protects against neurotoxic insults and up-regulates nAChRs, properties likely involved in its therapeutic effects^15–18^. Connections between the cholinergic system and α-secretase-mediated APP processing is gradually emerging. Several studies have shown that AChE inhibitors modify APP processing in favour of the non-amyloidogenic pathway in various systems, from SH-SY5Y cells to aged AD model mice^19–24^, but the underlying mechanism remains unclear.

In this study, we examined the effects of donepezil on APP processing in primary cortical neurons. We also identified SNX33 as a downstream factor up-regulated by donepezil treatment and examined its role in donepezil-dependent modification of APP processing.

## Results

### Donepezil up-regulates SNX33 expression

**Figure 1:**
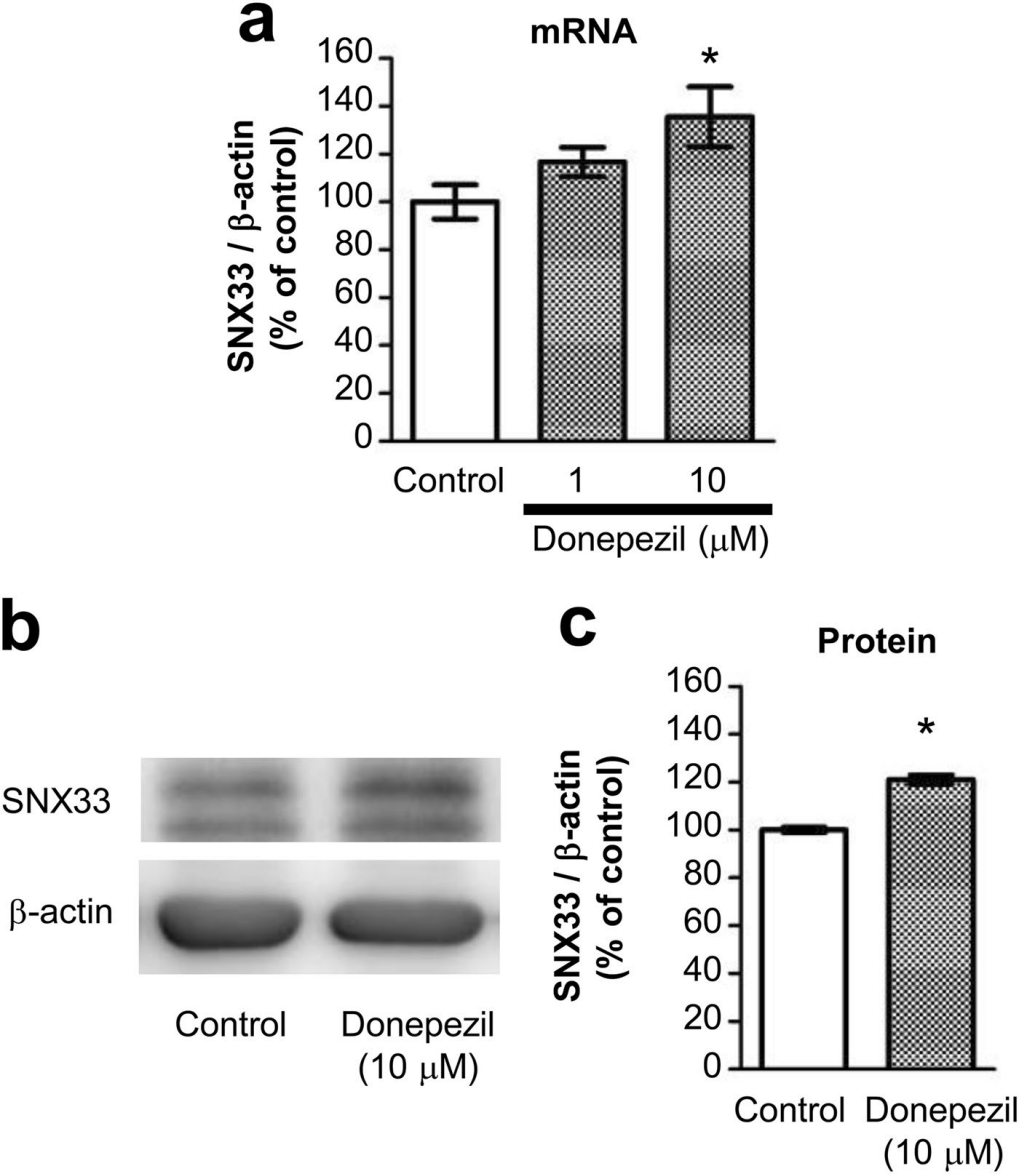
Effect of donepezil on SNX33 expression in primary cortical cells. (**a**) Cells were treated with 10 μM donepezil (1) for 24 h. The relative mRNA level of SNX33/β-actin was determined by RT-qPCR. *P < 0.05 compared with control cells. Data are expressed as mean ± SEM of n = 4 independent observations. (**b**,**c**) Cells were treated with 10 μM donepezil for 48 h. Cell lysate was subjected to western blotting. Representative images of western blotting analysis (**b**) and quantification of SNX33/β-actin ratio (**c**) are shown. ***P < 0.001 compared with control cells. Data are expressed as mean ± SEM of n = 3 independent observations. SNX33, sorting nexin protein 33; mRNA, messenger ribonucleic acid; RT-qPCR, real-time quantitative polymerase chain reaction.

In this study, to examine the mechanism involved in the neuroprotective effects observed by donepezil and galantamine treatment in primary cortical neurons, we initially screened for genes whose expression levels are up-regulated by these two drugs. Among candidate genes identified by microarray analysis of transcripts obtained from primary cortical neurons treated with donepezil or galantamine for 24 h, we focused on the up-regulation of SNX33 expression because the SNX family is believed to be involved in endomembrane transport of various transmembrane proteins, including APP^11^. In particular, SNX33 modulates APP endocytosis and APP cleavage by α-secretase, and the rate of APP endocytosis is a major control factor in APP cleavage by α-secretase^12^. qPCR examination of whether donepezil treatment under conditions previously been associated with neuroprotection up-regulates SNX33 transcription in primary cortical neurons demonstrated that donepezil-treated cells show a concentration-dependent increase in SNX33 mRNA levels compared with untreated control cells (Fig. 1a). In addition, western blotting performed to examine SNX33 expression showed that donepezil-treated cells have significantly increased SNX33 expression compared with untreated control cells (Fig. 1b,c). These results confirmed that donepezil treatment up-regulates SNX33 expression in primary cortical neurons.

### SNX33 is up-regulated in primary cortical neurons

**Figure 2:**
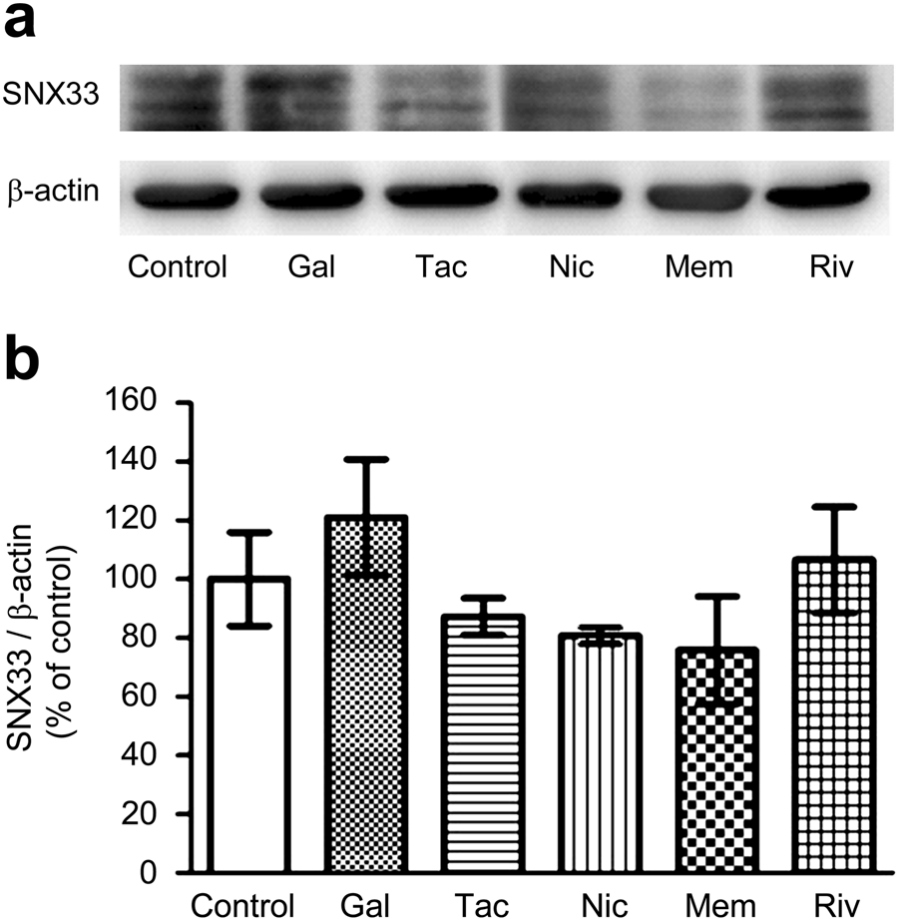
Effect of AD-related drugs on SNX33 expression in primary cortical cells. Cells were treated with 10 μM galantamine (Gal), 10 μM tacrine (Tac), 10 μM rivastigmine (Riv), 10 μM nicotine (Nic) and 10 μM memantine (Mem) for 48 h. Cell lysate was subjected to western blotting. Representative images of western blotting analysis (**a**) and quantification of SNX33/β-actin ratio (**b**) are shown. The grouping of blots was cropped from different gels in the same image. Data are expressed as mean ± SEM of n = 4–7 independent observations. AD, Alzheimer’s disease; SNX33, sorting nexin protein 33.

Treatment with galantamine and rivastigmine resulted in a slight increase in SNX33 expression, but statistical analysis did not support the significance of this increase (Fig. 2a,b). Treatment with tacrine, nicotine and memantine did not result in an increase in SNX33 expression. Therefore, under the treatment condition used in this study, we observed an increase in SNX33 expression with donepezil treatment. In subsequent experiments, we focused on the effects of donepezil treatment on primary cortical neurons.

### Donepezil treatment modulates APP processing in primary cortical neurons

**Figure 3:**
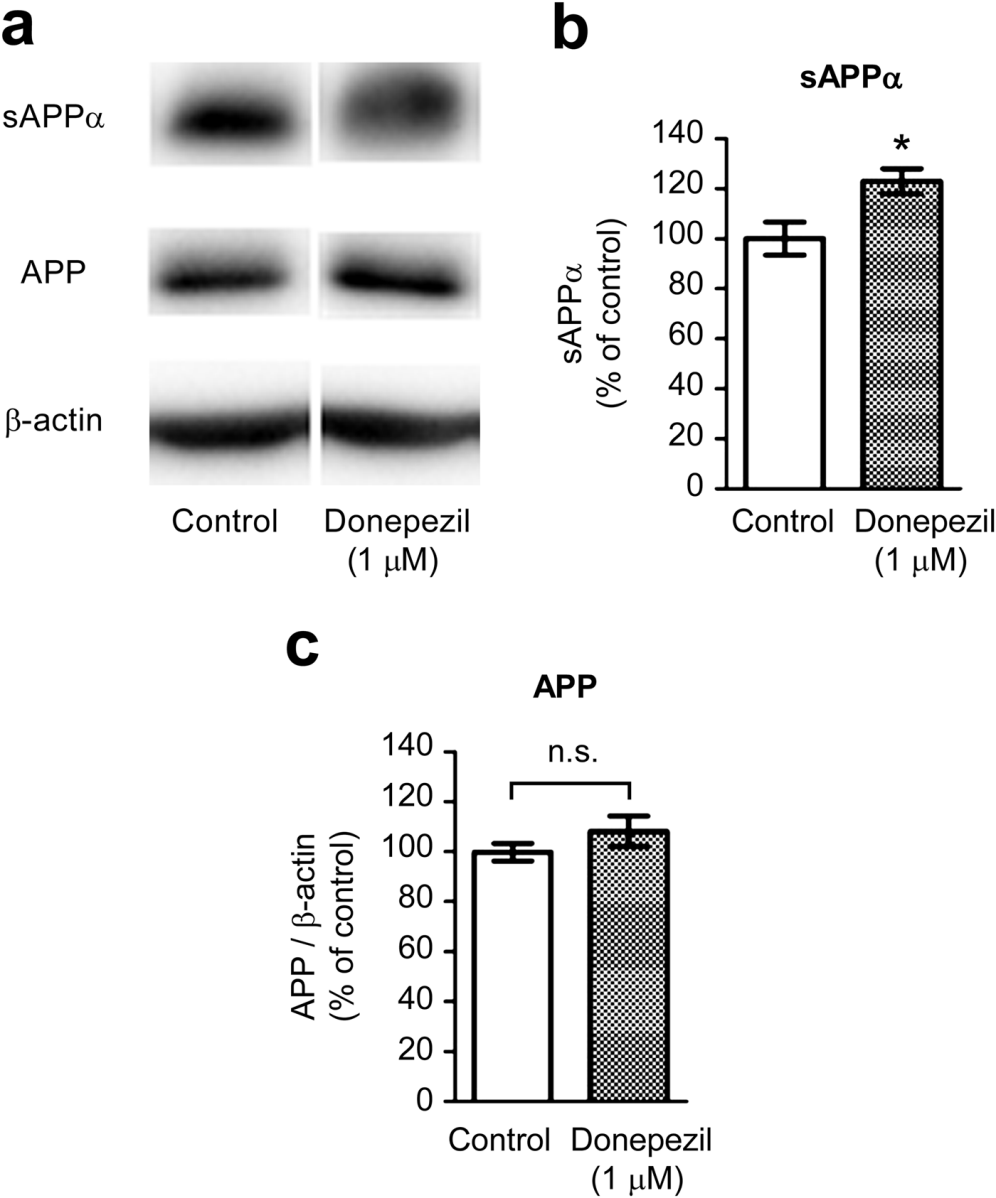
Effects of donepezil on non-amyloidogenic processing of APP in primary cortical cells. Cells were treated with 1 μM donepezil for 72 h. Cell lysate and culture media were subjected to western blotting. Representative images of western blotting analysis (**a**) and quantification of sAPPα (**b**) and APP/β-actin ratio (**c**) are shown. The grouping of blots was cropped from different parts of the same gel. *P < 0.05 compared with control cells. n.s., not significant. Data are expressed as mean ± SEM of n = 6 independent observations. APP, amyloid precursor protein.

**Figure 4:**
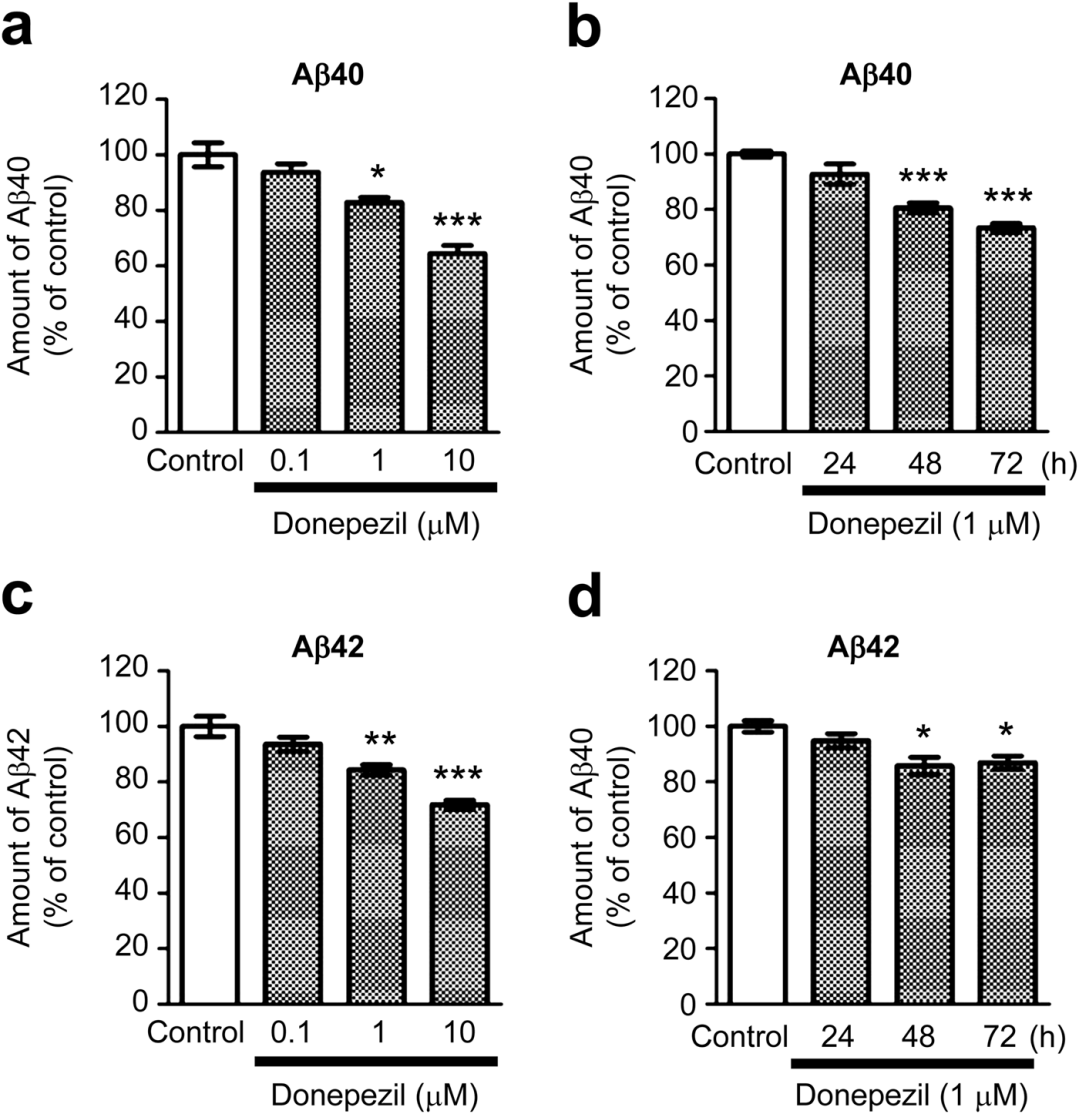
Effects of donepezil on Aβ content in culture media of primary cortical cells. Cells were treated with 0.1, 1 and 10 μM donepezil for 72 h (**a,b**) or with 1 μM donepezil for 24, 48 and 72 h (**c,d**). The amount of Aβ40 (**a,c**) and Aβ42 (**b,d**) in culture media were measured by ELISA. *P < 0.05; **P < 0.01; ***P < 0.001 compared with control cells. Data are expressed as means ± SEM of n = 3 independent observations. Aβ, amyloid β; ELISA, enzyme-linked immunosorbent assay.

Donepezil-treated cells showed a significant increase in sAPPα levels compared with untreated control cells (Fig. 3a,b). Consistent with the role of SNX33 in regulating the endocytotic transport of APP (the precursor of sAPPα) but not its expression^12^, we did not observe a significant increase in APP levels between donepezil-treated cells and untreated control cells (Fig. 3a,c). Further examination of the effects of donepezil on endocytic APP processing by observing Aβ levels in the culture media of primary culture neurons by enzyme-linked immunosorbent assay (ELISA) using Aβ40- and Aβ42-specific antibodies showed that the Aβ40 level in the culture media decreased significantly in a concentration- and time-dependent manner in donepezil-treated cells (Fig. 4a,b). The Aβ42 level also decreased significantly but after 48 h (Fig. 4c,d).

### Nicotinic and muscarinic AChR antagonists do not affect donepezil-induced modulation of APP shedding

**Figure 5:**
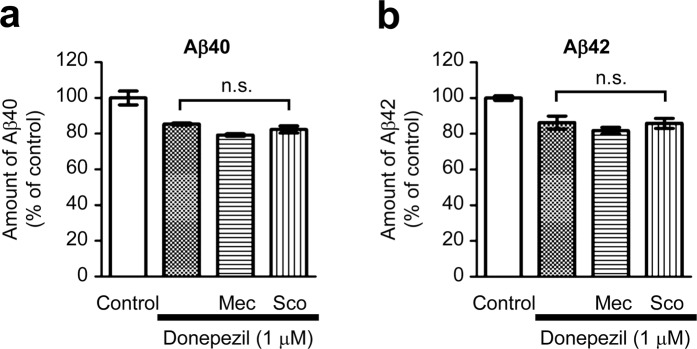
Involvement of nicotinic and muscarinic AChRs in the Aβ-reducing effect of donepezil in primary cortical cells. Cells were treated with 10 μM neuronal nAChR antagonist mecamylamine (Mec) or 10 μM mAChR antagonist scopolamine (Sco) simultaneously with 1 μM donepezil for 72 h. The amounts of Aβ40 (**a**) and Aβ42 (**b**) in culture media were measured by ELISA. n.s., not significant. Data are expressed as mean ± SEM of n = 3 independent observations. AChR, acetylcholine receptor; nAChR, nicotinic acetylcholine receptor; ELISA, enzyme-linked immunosorbent assay.

Our previous studies have shown that donepezil treatment up-regulates phosphoinositide 3-kinase (PI3K) signalling through activation of nAChRs^25–27^. Because PI3K signalling is involved in a multitude of cellular responses, including regulation of the expression of various downstream genes, donepezil might up-regulate SNX33 transcription, and thereby modulate APP cleavage via the nAChR-PI3K signalling pathway. Analysis of the effects of nicotinic and muscarinic AChR antagonists on donepezil-dependent decrease of Aβ40 and Aβ42 levels in the culture media by ELISA showed that simultaneous treatment with donepezil and either mecamylamine or scopolamine does not significantly alter the Aβ40 and Aβ42 levels in the culture media (Fig. 5a,b). Therefore, regulation of SNX33 expression by donepezil might depend on mechanisms independent of the nAChR-PI3K pathway.

### Target-specific morpholino oligos can suppress SNX33 expression in primary cortical cells

**Figure 6:**
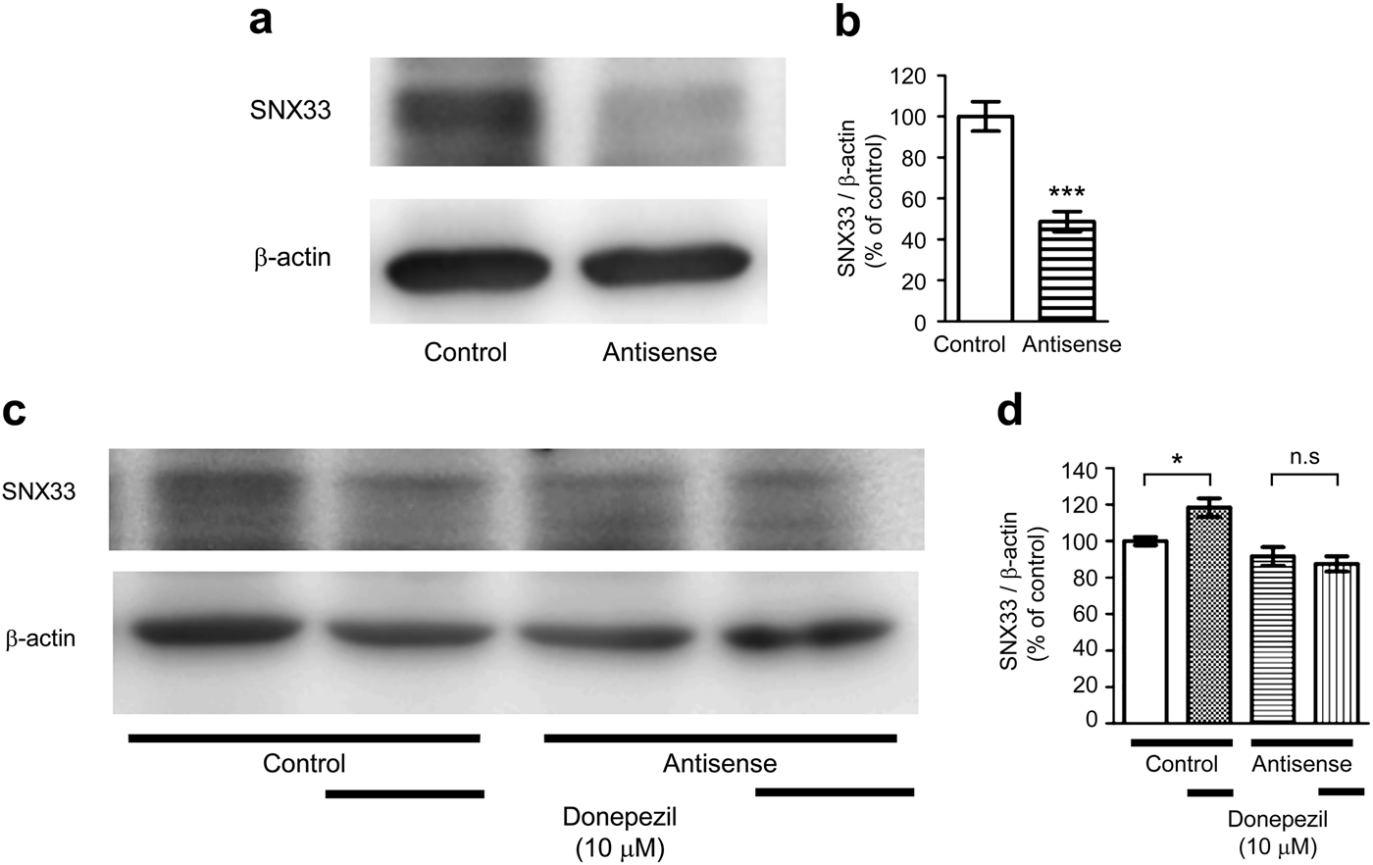
Involvement of SNX33 in effects of donepezil in primary cortical cells. (**a,b**) Effect of antisense MOs on SNX33 expression. Cells were treated with 1 μM control or antisense MOs, in addition to 6 μL/mL of Endo Porter (EP) for 24 h, and 48 h later, the cells were harvested. Cell lysate was subjected to western blotting. Representative images of western blotting analysis (**a**) and quantification of SNX33/β-actin ratio (**b**) are shown. ***P < 0.001 compared with control cells. n.s., not significant. Data are expressed as mean ± SEM of n = 5 independent observations. (**c,d**) Effect of SNX33 knockdown on donepezil-induced SNX33 expression. Cells were pretreated with MOs and EP for 24 h prior to 10 μM donepezil treatment for 48 h. Cell lysate was subjected to western blotting. Representative images of western blotting analysis (**b**) and quantification of SNX33/β-actin ratio (**c**) are shown. *P < 0.05; n.s., not significant. Data are expressed as mean ± SEM of n = 12–18 independent observations. SNX33, sorting nexin protein 33; MO, morpholino oligo.

Primary neurons transfected with SNX33 morpholino oligos (MOs) showed significantly lower SNX33 expression than cells transfected with control MOs (Fig. 6a,b), suggesting that target-specific MOs can inhibit SNX33 expression. Cells transfected with control MOs showed a significant increase in SNX33 expression by donepezil treatment, but in cells transfected with SNX33 MOs, donepezil treatment did not significantly increase SNX33 expression (Fig. 6c,d). These results indicated that transfection of target-specific MOs effectively suppresses gene expression of SNX33 in primary cortical cultures and prompted us to investigate the role of SNX33 in donepezil-dependent modulation of APP processing.

### SNX33 is required for modulation of APP processing by donepezil treatment

**Figure 7:**
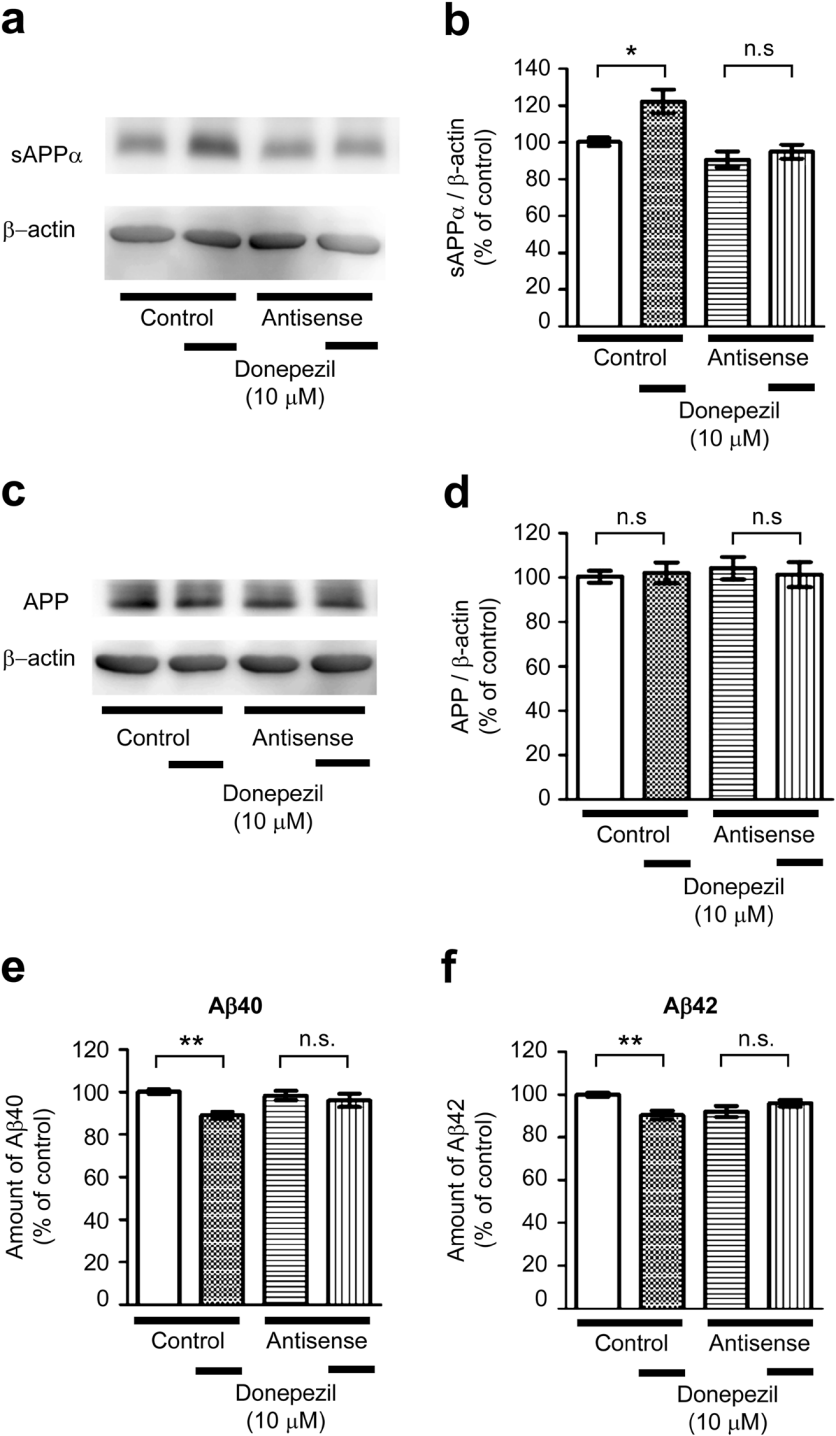
Involvement of SNX33 in effects of donepezil on APP processing and Aβ content in culture media in primary cortical cells. (**a**–**d**) Effect of SNX33 knockdown on non-amyloidogenic APP processing in primary cortical cells. Cells were treated with 1 μM control or antisense MOs, in addition to 6 μL/mL of Endo Porter (EP) for 24 h, washed and treated with donepezil for 48 h. After donepezil treatment, cell lysate and culture media were subjected to western blotting. Representative images of western blotting analysis (**a**) and quantification of sAPPα/β-actin ratio (**b**) are shown. *P < 0.05; n.s., not significant. Data are expressed as mean ± SEM of n = 11–15 independent observations. (c,d) Representative images of western blotting analysis (**c**) and quantification of APP/β-actin ratio (**d**) are shown. n.s., not significant. Data are expressed as mean ± SEM of n = 16–18 independent observations. (**e,f**) Effect of SNX33 knockdown on donepezil-induced Aβ reduction. Cells were pretreated with MOs and EP for 24 h prior to treatment with 10 μM donepezil for 48 h. The amounts of Aβ40 (**e**) and Aβ42 (**f**) in culture media were measured by ELISA. **P < 0.01; ***P < 0.001; n.s., not significant. Data are expressed as mean ± SEM of n = 12 (**e**) or n = 13 (**f**) independent observations. SNX33, sorting nexin protein 33; MO, morpholino oligo; ELISA, enzyme-linked immunosorbent assay; APP, amyloid precursor protein.

In control MO-transfected cells, donepezil treatment resulted in a significant increase in the sAPPα levels (Fig. 7a,b). However, in SNX33 MO-transfected cells, the increase in sAPPα levels was not significant. Because this lack of increase in sAPPα levels may be owing to decrease in APP levels, we examined APP levels in control and SNX33 MO-transfected cells. Results showed no significant change in APP levels between control and SNX33 MO-transfected cells or between donepezil-treated and untreated cells (Fig. 7c,d). In addition, although donepezil treatment led to a significant decrease in Aβ40 and Aβ42 levels in control MO-transfected primary cortical neurons (Fig. 7e,f), it did not significantly decrease Aβ40 and Aβ42 levels in SNX33 MO-transfected cells.

### Cell surface expression of APP increases by donepezil treatment

**Figure 8:**
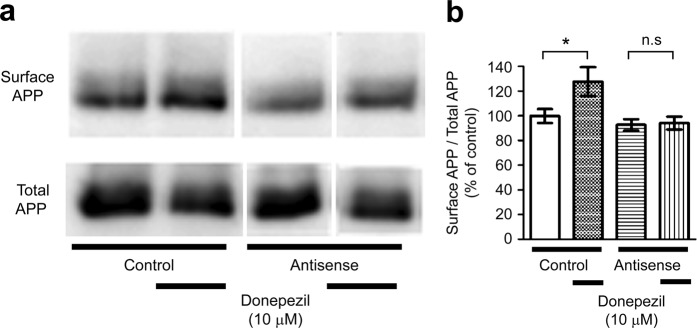
Effects of donepezil on cell membrane surface expression of APP in primary cortical cells. (**a,b**) Cells were treated with 1 μM donepezil for 48 h. Cell membrane surface proteins were biotinylated and affinity precipitated by immobilised streptavidin beads. Collected cell membrane surface proteins and total cell lysate were subjected to western blotting. (**a**) Representative images of APP in affinity-precipitated fraction (surface APP) and in total cell lysate (total APP) are shown. (**b**) Quantification of surface APP/total APP ratio is shown. The grouping of blots was cropped from different parts of the same gel. *P < 0.05; n.s., not significant. Data are expressed as mean ± SEM of n = 8–10 independent observations. APP, amyloid precursor protein.

Analysis of APP expression at the cell surface showed that in control MO-transfected cells, APP levels on the cell surface increased with donepezil treatment (Fig. 8a,b). However, we did not observe an increase in APP levels on the cell surface in SNX33 MO-transfected cells. (Fig. 8a,b).

## Discussion

In this study, we identified SNX33 as a downstream factor up-regulated by donepezil treatment. We also investigated the effects of donepezil treatment on APP processing in primary cortical neurons and role of SNX33 in donepezil treatment-induced effects.

Our results are consistent with the notion that donepezil modifies APP processing via up-regulation of SNX33 expression. Donepezil up-regulated sAPPα and surface APP levels but not total APP levels. Further, donepezil significantly decreased Aβ levels in culture media. Because cleavage by α-secretase, which generates sAPPα, occurs within the Aβ sequence and precludes Aβ generation, the effects of donepezil are consistent with the regulation of APP processing by donepezil. The effects of donepezil were reversed upon inhibition of the expression of SNX33, whose function is to modify APP processing through regulation of endocytotic transport of APP^12^. These results are consistent with an important role SNX33 plays in donepezil-dependent modification of APP processing.

According to a straightforward model, donepezil treatment increases SNX33 expression, which reduces the rate of APP endocytosis, and leads to increased APP levels at the cell membrane surface, where APP is cleaved by α-secretase, thus decreasing Aβ generation. However, Zimmerman *et al*. ^20^ showed that donepezil treatment increases the cell membrane levels of constitutive α-secretases of APP, ADAM10 and ADAM17^20^. Ebsen and colleagues showed that the SH3 domain of SNX33 interacts with ADAM10^28^. This raises the possibility that the donepezil-dependent increase in sAPPα and decrease in Aβ are owing to the SNX33-dependent increase of ADAM10 and ADAM17 levels and their activity or stability. Further studies are required to uncover how donepezil regulates APP processing via up-regulation of SNX33 expression.

In this study, we were unable to identify the mechanism responsible for the up-regulation of SNX33 expression. Our previous studies have reported that donepezil activates the PI3K signalling pathway through nAChR activation and that donepezil regulates at least one of its downstream effector genes, α7-nAChR, through the nAChR-PI3K signalling pathway^25,29^. Our initial assumption was that SNX33 expression is up-regulated via the nAChR-PI3K signalling pathway. However, we observed that neither nicotinic nor muscarinic AChR antagonists can reverse the effects of donepezil treatment. These results are consistent with those of a previous study, in which the effects of atropine and bungarotoxin (muscarinic and nicotinic receptor antagonists, respectively) were tested on the donepezil-dependent increase of sAPPα in SH-SY5Y cells^20^. Conversely, the Aβ-decreasing effect of galantamine, an allosteric potentiating ligand of nAChRs^30^, is blocked by methyllycaconitine (an α7-nAChR antagonist)^21,22^, bungarotoxin, atropine and mecamylamine^21^, suggesting that AChRs play a role in galantamine-dependent modulation of APP processing. It is still unclear whether rivastigmine modifies APP processing through AChRs, but stimulation of muscarinic AChRs has been shown to increase sAPPα release^19^. Further analysis is required to understand the mechanism leading to SNX33 up-regulation and uncover similarities, if any, between donepezil- and galantamine-dependent modifications of APP processing. Such mechanism may become a therapeutic target to control Aβ levels via regulation of APP processing.

The effects of AD drugs on suppression of Aβ production have also been tested *in vivo*. Dong and colleagues studied a Tg2576 AD mouse model and showed that donepezil treatment at higher concentrations decreases soluble Aβ protein level and Aβ accumulation compared with lower concentrations^31^. A similar decrease in soluble Aβ protein level, along with improvements in cognitive performance, was observed upon donepezil administration to aged AD model mice with fully developed amyloid pathology^32^. Wu *et al*.^24^ showed that galantamine treatment reduces the total area of amyloid load within the hippocampus in aged APP/PS1 mice^24^. In addition, rivastigmine treatment increases sAPPα and decreases Aβ40 levels in primary embryonic rat cortical cultures^23^. However, the mechanism altering sAPPα and Aβ levels remains unknown. It should also be noted that one study reported that donepezil administration to human patients does not result in a significant decrease in brain Aβ accumulation^33^. On the contrary, a large-scale study has shown that donepezil delays hippocampal atrophy in patients suspected to have prodromal AD, which supports the neuroprotective effect of donepezil^34^. These results indicate that donepezil, galantamine and rivastigmine seem to share the potential to modulate APP processing, although the underlying mechanism is still unclear. Our study might contribute to a further understanding of the phenomenon. Because endocytic transport is among the earliest to display neuropathological abnormalities during early-stage AD and is believed to precede Aβ deposition^35,36^, a new AD therapeutic strategy towards control of APP processing via action of SNX family proteins could be promising. Whether there are common mechanisms for APP modification by donepezil, galantamine and rivastigmine is yet to be examined. It would be interesting to investigate whether the expression of other SNX family proteins is up-regulated by galantamine and rivastigmine treatment and whether such proteins are responsible for a decrease in Aβ production.

## Conclusion

In conclusion, we identified a downstream factor of donepezil, SNX33, an endosomal trafficking protein known to regulate processing of APP. Our results indicate that donepezil regulates endocytic trafficking of APP via up-regulation of SNX33 expression.

## Methods

### Materials

We purchased Eagle’s minimal essential medium (EMEM) from Nissui Pharmaceutical (Tokyo, Japan). Fetal bovine serum (FBS) and horse serum were purchased from JRH Biosciences (Lenexa, KS, USA). The drugs used in this study and their sources were as follows: mecamylamine-HCl and (2)-scopolamine-HBr from Sigma (St. Louis, MO, USA); donepezil hydrochloride[(6)-2-[(1-benzylpiperidin-4-yl)methyl]-5,6-dimethoxy-indan-1-one monohydrochloride; E2020] from Eisai (Tsukuba, Japan); galanthamine hydrobromide, tacrine hydrochloride, memantine hydrochloride and rivastigmine tartrate from Sigma and (−)-nicotine tartrate from ICN Biomedicals, Inc. (Costa Mesa, CA, USA). The stock solutions of drugs were dissolved in EMEM immediately before experiments. Anti-SNX33 antibody was kindly provided by Dr. Stefan F. Lichtenthaler (Technical University of Munich, Germany). Anti-sAPPα antibody (Code #28055) was purchased from Immuno-Biological Laboratories (IBL; Gunma, Japan). Anti-APP (Code #A8717) and Anti-β-actin (Code #A1978; Clone AC-15) antibodies were purchased from Sigma (St. Louis, MO, USA). Anti-rabbit (Code #NA934) or anti-mouse (Code #NA931) immunoglobulin [IgG] horseradish peroxidase-linked whole antibodies were purchased from GE Healthcare (Buckinghamshire, UK).

### Cell culture

The animal study was carried out in accordance with the guidelines of the Kyoto University Animal Experimentation Committee and the Japanese Pharmacological Society. This study was approved by Kyoto University Animal Experimentation Committee. We prepared rat primary cortical cultures from Wistar/ST rat embryos (17–19 days of gestation), as described previously^37^. Briefly, single cells dissociated from the entire cerebral cortex of fetal rats were plated on 0.1% polyethyleneimine-coated plates. Next, cultures were maintained in EMEM supplemented with 10% heat-inactivated FBS (1–7 days after plating) or 10% heat-inactivated horse serum (8–12 days after plating), 2 mM glutamine, total 11 mM glucose, 24 mM NaHCO3 and 10 mM 4-(2-hydroxyethyl)-1-piperazineethanesulfonic acid at 37 °C in a humidified 5% CO2 atmosphere. During 6–9 days *in vitro*, the cells were treated with 10 μM arabinosylcytosine, and at 10–12 days *in vitro*, the cells were subjected to experiments.

### Real-time PCR

Real-time PCR was performed basically as previously described^38^. We washed the treated cells twice with cold phosphate-buffered saline (PBS) and extracted total ribonucleic acid (RNA) using the Reliaprep™ RNA cell miniprep system (Promega Corporation, Fitchburg, WI, USA). Next, we performed reverse transcription (RT) of messenger RNA (mRNA) using the Goscript™ RT system (Promega Corporation). Finally, we performed real-time quantitative polymerase chain reaction (RT-qPCR) using the GoTaq™ qPCR master mix (Promega Corporation) and optimised the PCR conditions using a TaKaRa PCR thermal cycler Dice (TaKaRa Bio, Inc., Shiga, Japan). The primer sequences used were as follows: β-actin forward, 5′-GGAGATTACTGCCCTGGCTCCTA-3′, β-actin reverse, 5′-GACTCATCGTACTCCTGCTTGCTG-3′, SNX33 forward, 5′-GCGAGACCTCACTGGACGGCT-3′ and SNX33 reverse, 5′-TTGGTGCTGATGCCAGGACGA-3′.

### Western blotting

Western blotting was performed basically as previously described^39^. Culture media were collected, and the cells were washed twice with cold Tris-buffered saline, harvested with a cell scraper and lysed in a buffer containing 20 mM Tris, 25 mM β-glycerophosphate, 2 mM ethylene glycol-bis(β-aminoethyl ether)-N,N,N′,N′-tetraacetic acid, 1% Triton X-100, 1 mM phenylmethylsulfonyl fluoride, 1% aprotinin, 2 mM dithiothreitol and 1 mM vanadate on ice. Next, we sonicated lysates and centrifuged them at 15,000 rpm at 4 °C for 30 min. After normalisation of protein concentrations, we mixed the supernatants in equal amounts with a sample loading buffer. Protein was denatured by boiling for 5 min. Then, the samples were loaded onto a sodium dodecyl sulfate (SDS)-polyacrylamide gel, separated electrophoretically and transferred to a polyvinylidene fluoride (PVDF) membrane (Millipore, Bedford, MA, USA). To block non-specific protein binding, the PVDF membrane was incubated with 10 mM Tris-buffered saline containing 0.1% Tween-20 and 5% dehydrated skim milk. Then, the PVDF membrane was again incubated with primary antibodies (anti-SNX33, anti-sAPPα, anti-APP or anti-β-actin) and secondary antibodies (anti-rabbit or anti-mouse immunoglobulin [IgG] horseradish peroxidase-linked whole antibody [GE Healthcare, Buckinghamshire, UK]). Finally, we performed immunodetection with an enhanced chemiluminescence detection system (GE Healthcare). Band intensities were measured using the ImageJ software (National Institutes for Health, Bethesda, Maryland, USA). Original images of the gels are shown in the Supplemental Figures.

### ELISA

We collected the culture media and centrifuged them to discard debris. Aβ protein concentrations in the culture media were measured using Human/Ratβ amyloid (40) ELISA Kit Wako II (Code #294-64701) or Human/Rat β amyloid (42) ELISA Kit Wako, High-Sensitive (Code #292-64501) purchased from Fujifilm Wako Pure Chemical Corporation (Osaka, Japan). Microplate reader (Model 680; Bio-Rad Laboratories, Inc.) was used in accordance with the manufacturer’s instructions. In the Aβ40 ELISA kit, monoclonal antibodies BNT77 and BA27, which specifically detects Aβ(11–28) and the C-terminal portion of Aβ(x-40), respectably, were used. In the Aβ42 ELISA kit, monoclonal antibodies BNT77 and BC05, which detects Aβ(11–28) and the C-terminal portion of Aβ(x-42) were used, respectably.

### Introduction of morpholino oligos

We delivered 1 mM MOs; Genetools, Philomath, OR, USA) into the cells using the Endo Porter delivery reagent. The sequence of antisense MOs was as follows: 5′-CATGGCTAAGTTATCTGTTAGGTCA-3′. Standard Control Oligo, Classic was used as a negative control. The sequence of control MOs was as follows: 5′-CCTCTTACCTCAGTTACAATTTATA-3′.

### Preparation of cell membrane surface proteins

We prepared cell membrane surface proteins using the Pierce® Cell Surface Protein Isolation Kit (Thermo Fisher Scientific, MA, USA) according to the manufacturer’s instructions. Briefly, cells were washed twice with cold PBS. Then, the cell membrane surface was biotinylated by incubating the cells with PBS containing Sulfo-NHS-SS-Biotin. Next, cells were harvested with a cell scraper into a conical tube and centrifuged. The supernatant was then discarded, and cells were pipetted with Tris-buffered saline and centrifuged. The supernatant was again discarded. Cells were then lysed, sonicated and centrifuged at 15,000 rpm at 4 °C for 2 min. After normalisation of protein concentrations, we applied the supernatant to NeutrAvidin Agarose gel columns and incubated it for 1 h. Gels were washed thrice, and biotinylated surface proteins stuck to the gel were eluted by incubating the gel with a SDS-polyacrylamide gel electrophoresis sample buffer containing dithiothreitol for 1 h. Finally, total lysates and eluted surface proteins were subjected to western blotting.

### Statistical analysis

Data were expressed as mean ± standard error of the mean (SEM). The statistical significance of between-group differences was determined with the unpaired t-test or one-way analysis of variance, followed by Tukey’s test, using Prism software (GraphPad Software, San Diego, CA, USA). *P* < 0.05 was considered to be statistically significant.

## Supplementary information

Supplemental Figures
