# Supplementary material for: Donepezil modulates amyloid precursor protein endocytosis and reduction by up-regulation of SNX33 expression in primary cortical neurons
Source: Sci Rep. 2019 Aug 15;9:11922. doi: 10.1038/s41598-019-47462-4 (PMC6695423; doi:10.1038/s41598-019-47462-4)
Supplement: Supplementary file 1 — Supplemental Figures [file 41598_2019_47462_MOESM1_ESM.pdf]

Donepezil modulates amyloid precursor protein endocytosis and reduction by up-regulation of SNX33 expression in primary cortical neurons

Yuki Takada-Takatori[a*], Shota Nakagawa[b], Riko Kimata[a], Yousuke Nao[b], Yumiko Mizukawa[a], Tetsuro Urushidani[a], Yasuhiko Izumi[b,c], Akinori Akaike[b,d], Katsuharu Tsuchida[a], Toshiaki Kume[b,e]

[a]Faculty of Pharmaceutical Sciences, Doshisha Women's College, Kyoto 610-0395, Japan
[b]Department of Pharmacology, Graduate School of Pharmaceutical Sciences, Kyoto University, Kyoto 606-8501, Japan
[c]Laboratory of Pharmacology, Kobe Pharmaceutical University, Hyogo 658-8558, Japan
[d]Department of Pharmacology, Graduate School of Medicine, Wakayama Medical University, Wakayama 641-0012, Japan
[e]Department of Applied Pharmacology, Graduate School of Medical and Pharmaceutical Sciences, University of Toyama, Toyama 930-0194, Japan

Corresponding author*
Yuki Takada-Takatori
Faculty of Pharmaceutical Sciences, Doshisha Women's College, Kyoto 610-0395, Japan
E-mail address: ytakator@dwc.doshisha.ac.jp

Supplementary Information

1. Control
2. Donepezil

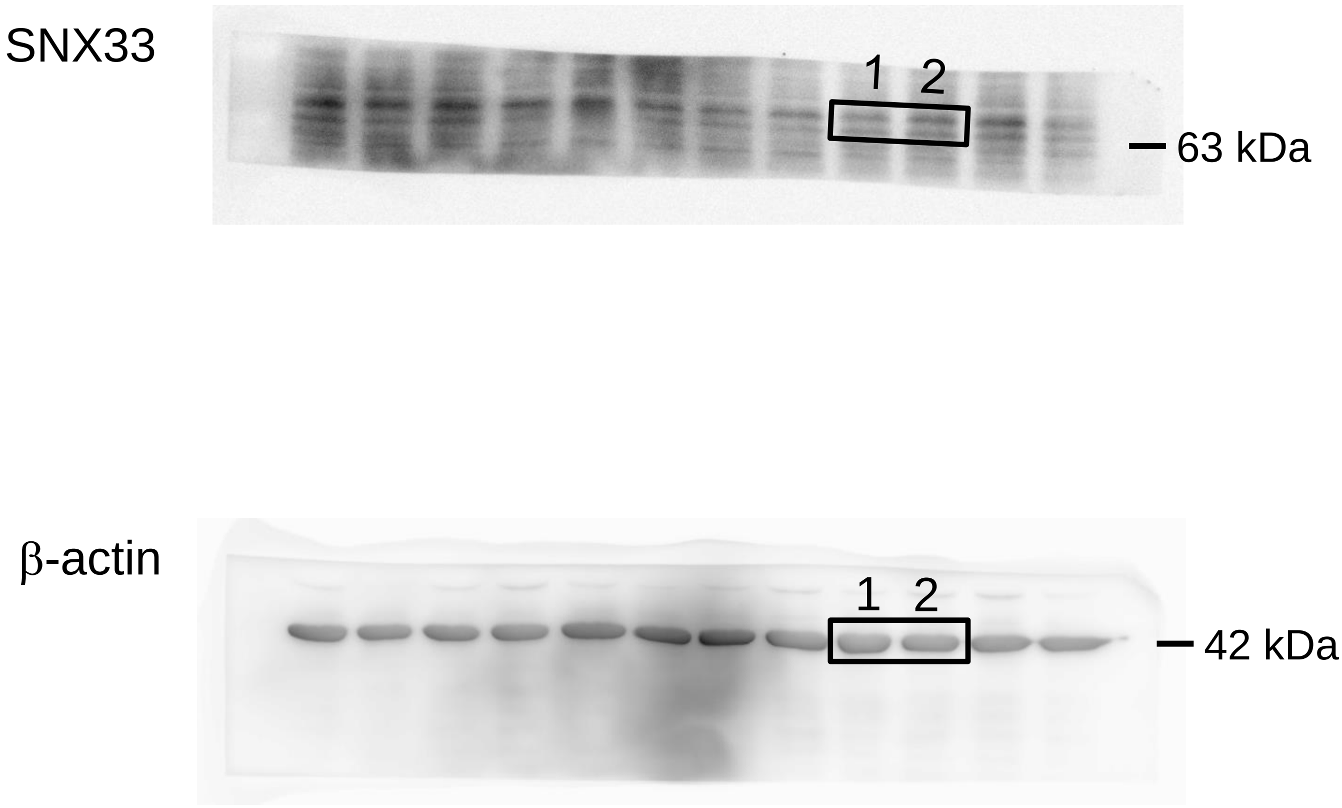

Supplemental figure S1. Original full length of blots for Fig. 1B. Boxes indicate areas shown in figure.

Cont: Control
Gal: Galantamine
Tac: Tacrine
Riv: Rivastigmine
Nic: Nicotine
Mem: Memantine

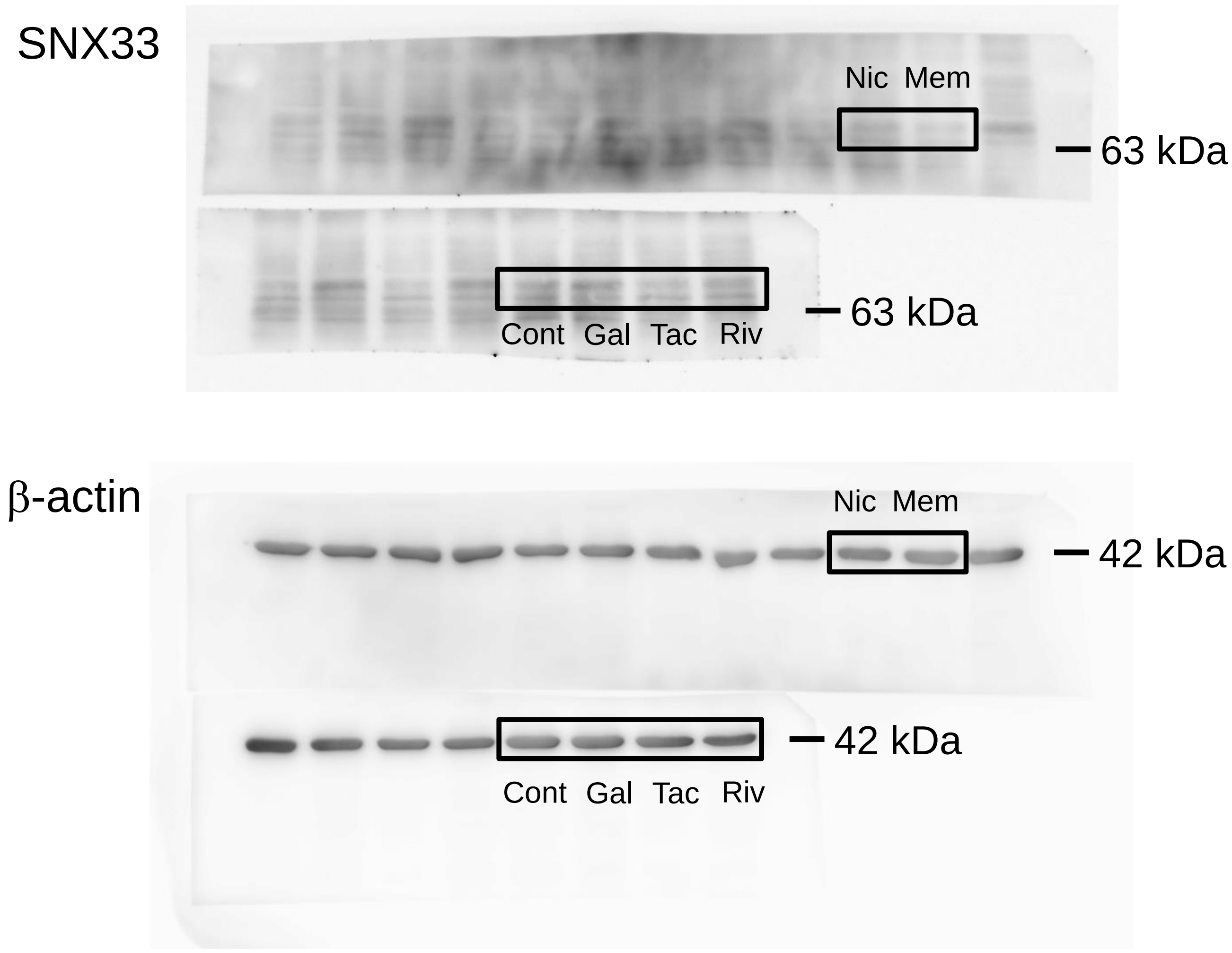

Supplemental figure S2. Original full length of blots for Fig. 2A. Boxes indicate areas shown in figure.

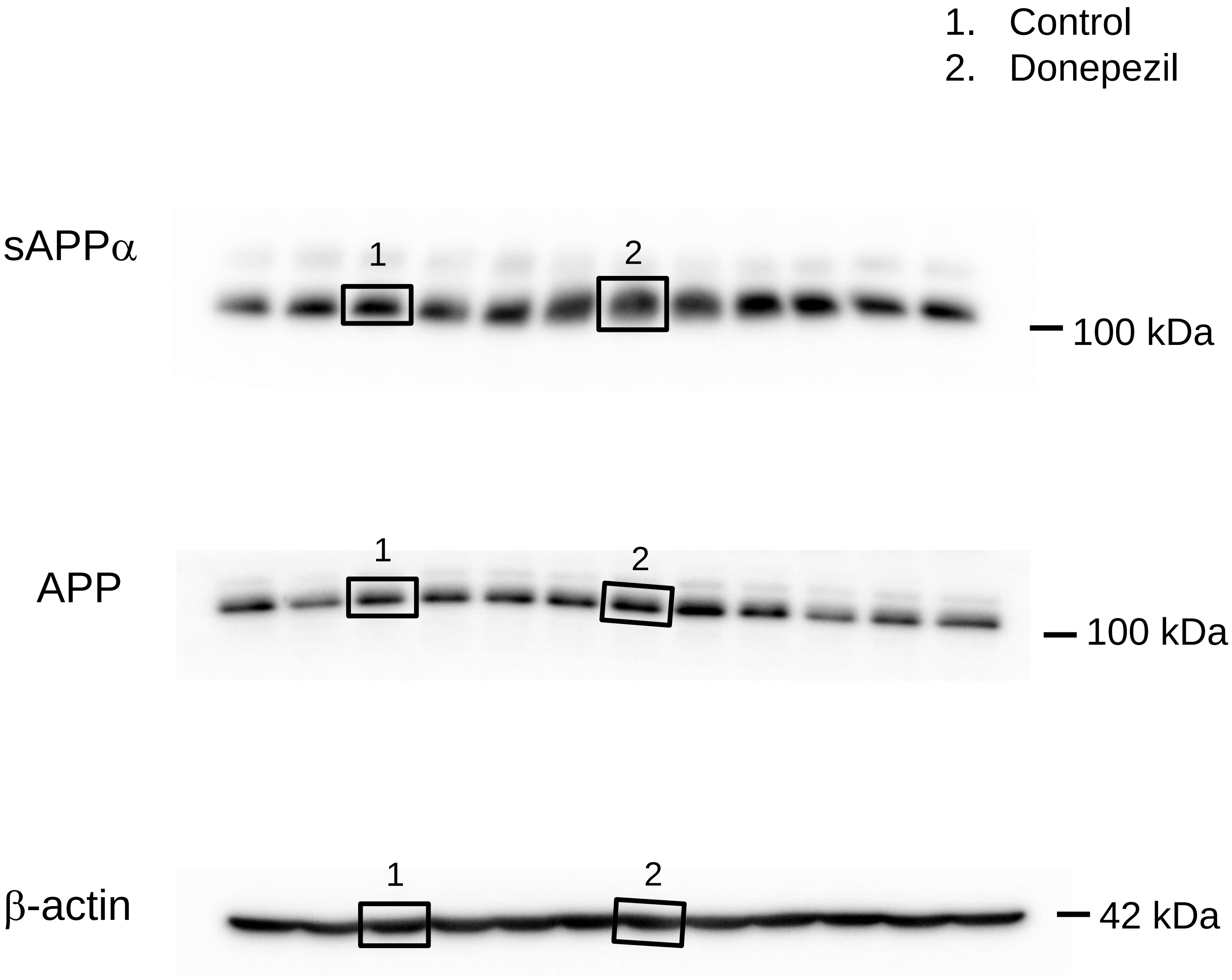

Supplemental figure S3. Original full length of blots for Fig. 3A. Boxes indicate areas shown in figure.

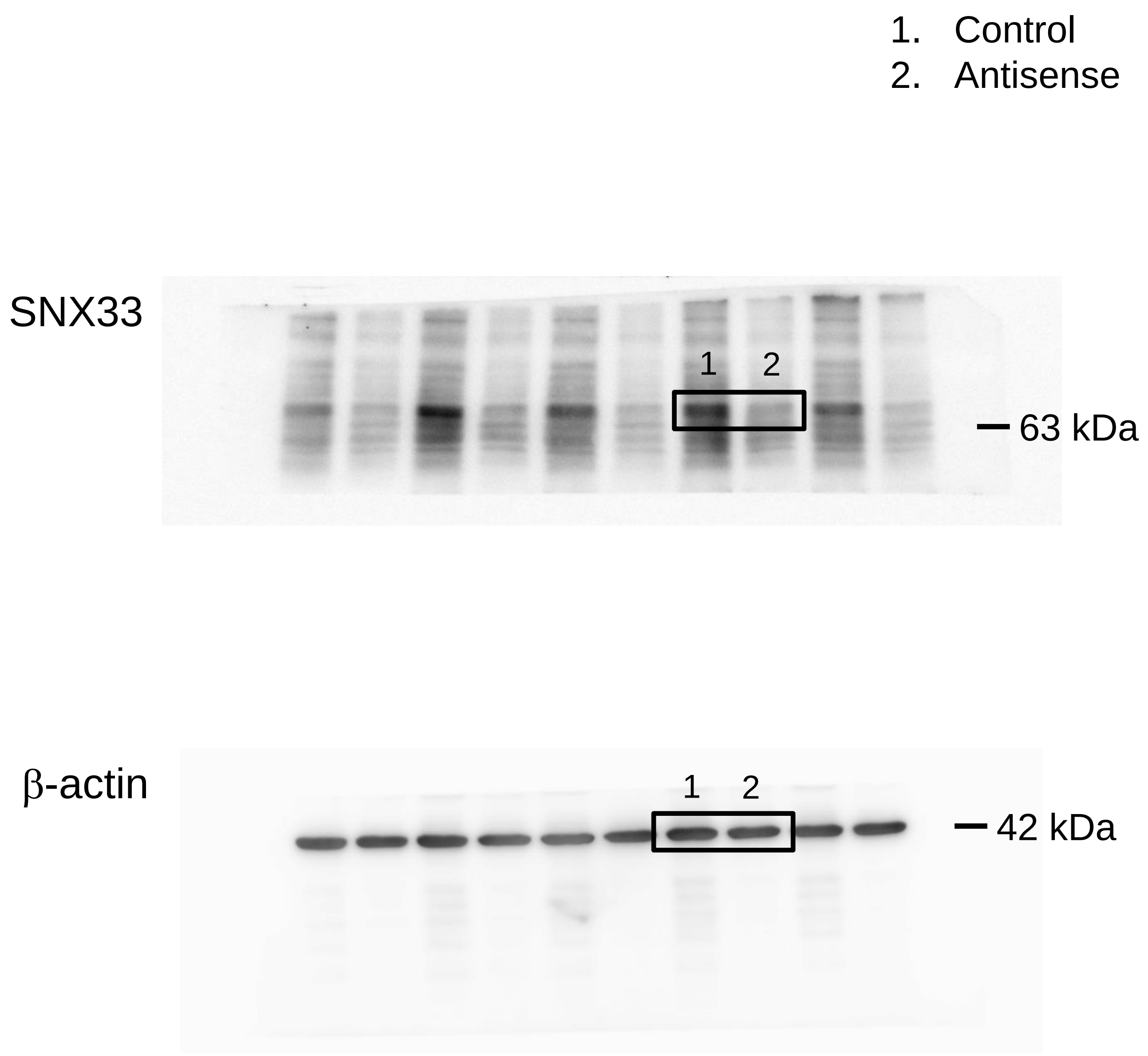

Supplemental figure S4. Original full length of blots for Fig. 6A. Boxes indicate areas shown in figure.

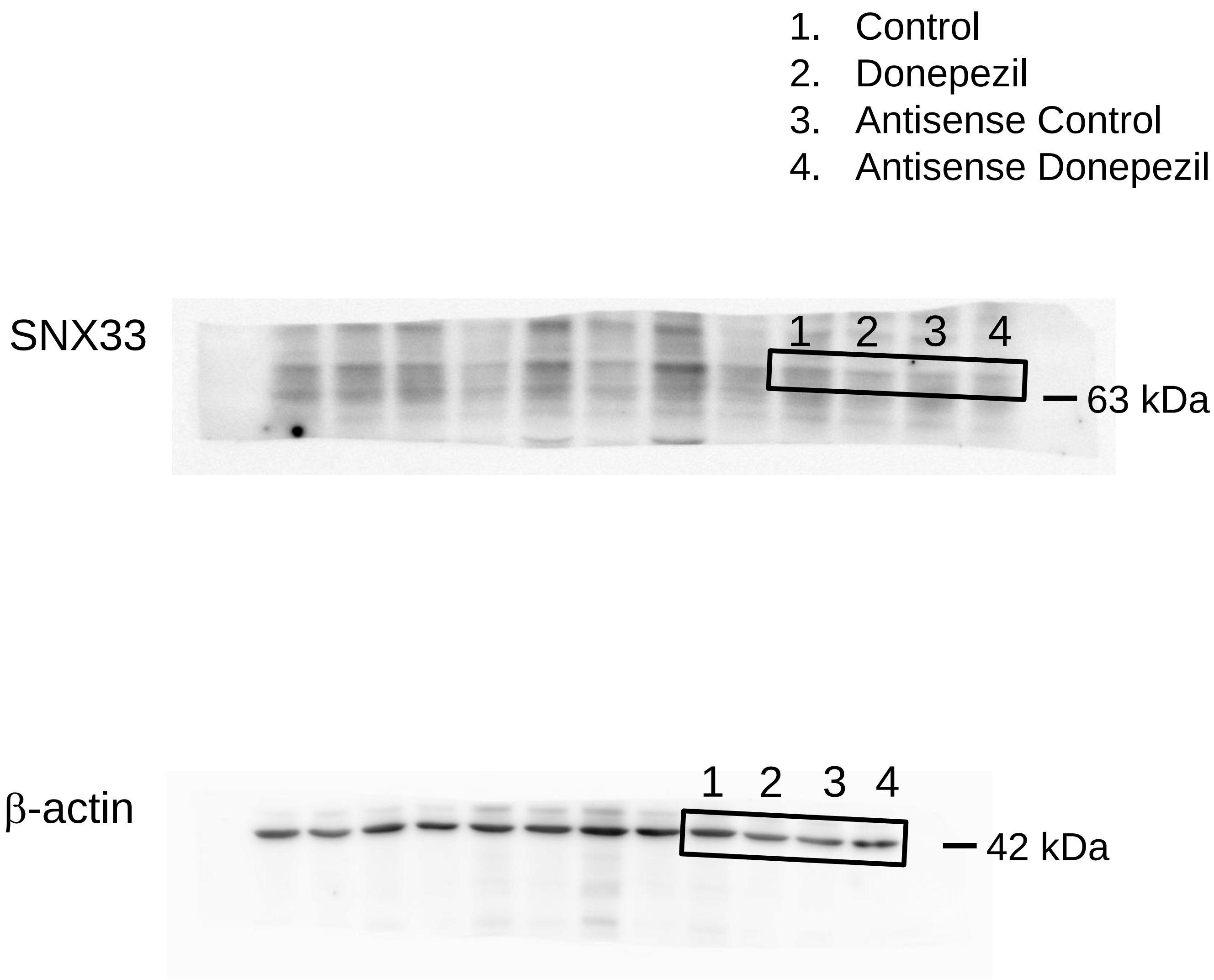

Supplemental figure S5. Original full length of blots for Fig. 6C. Boxes indicate areas shown in figure.

1. Control
2. Donepezil
3. Antisense Control
4. Antisense Donepezil

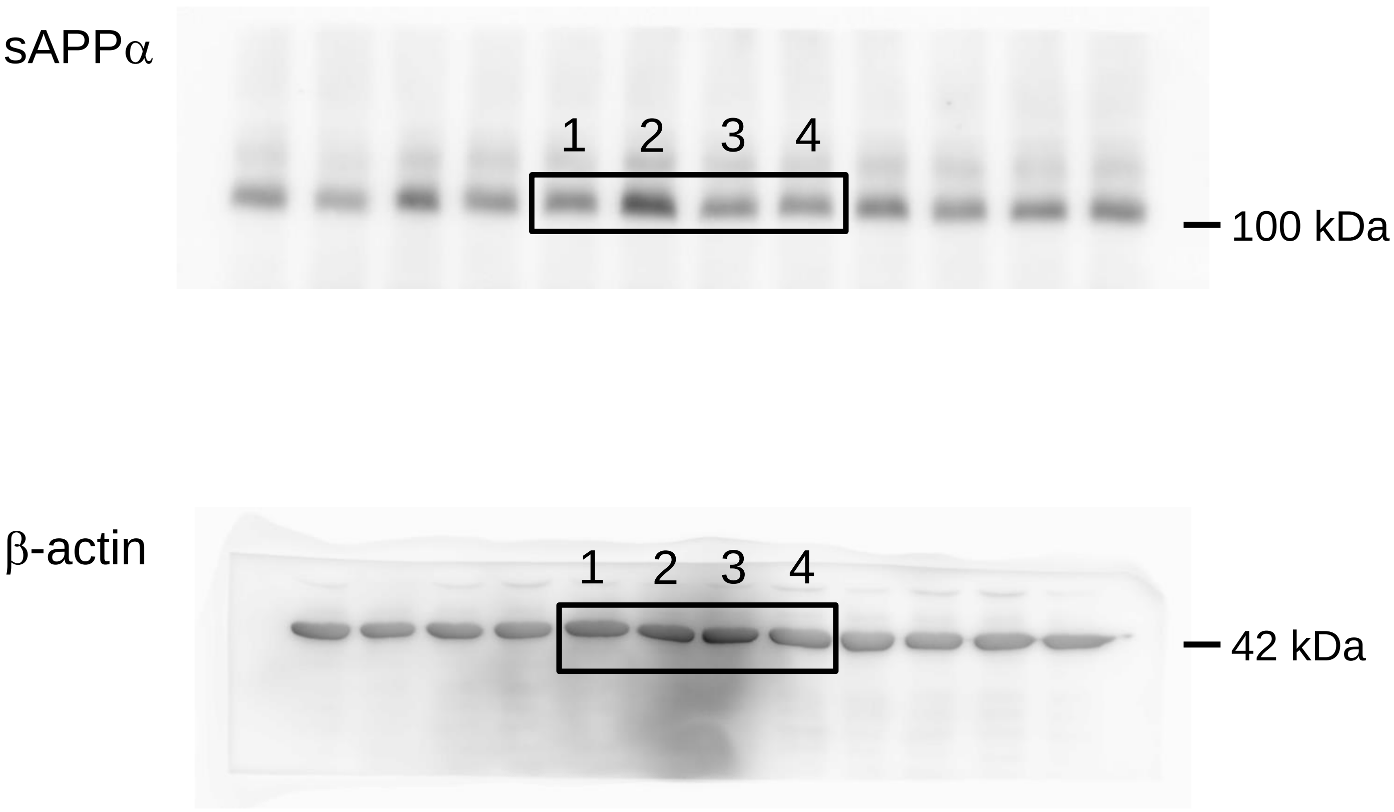

Supplemental figure S6. Original full length of blots for Fig. 7A. Boxes indicate areas shown in figure.

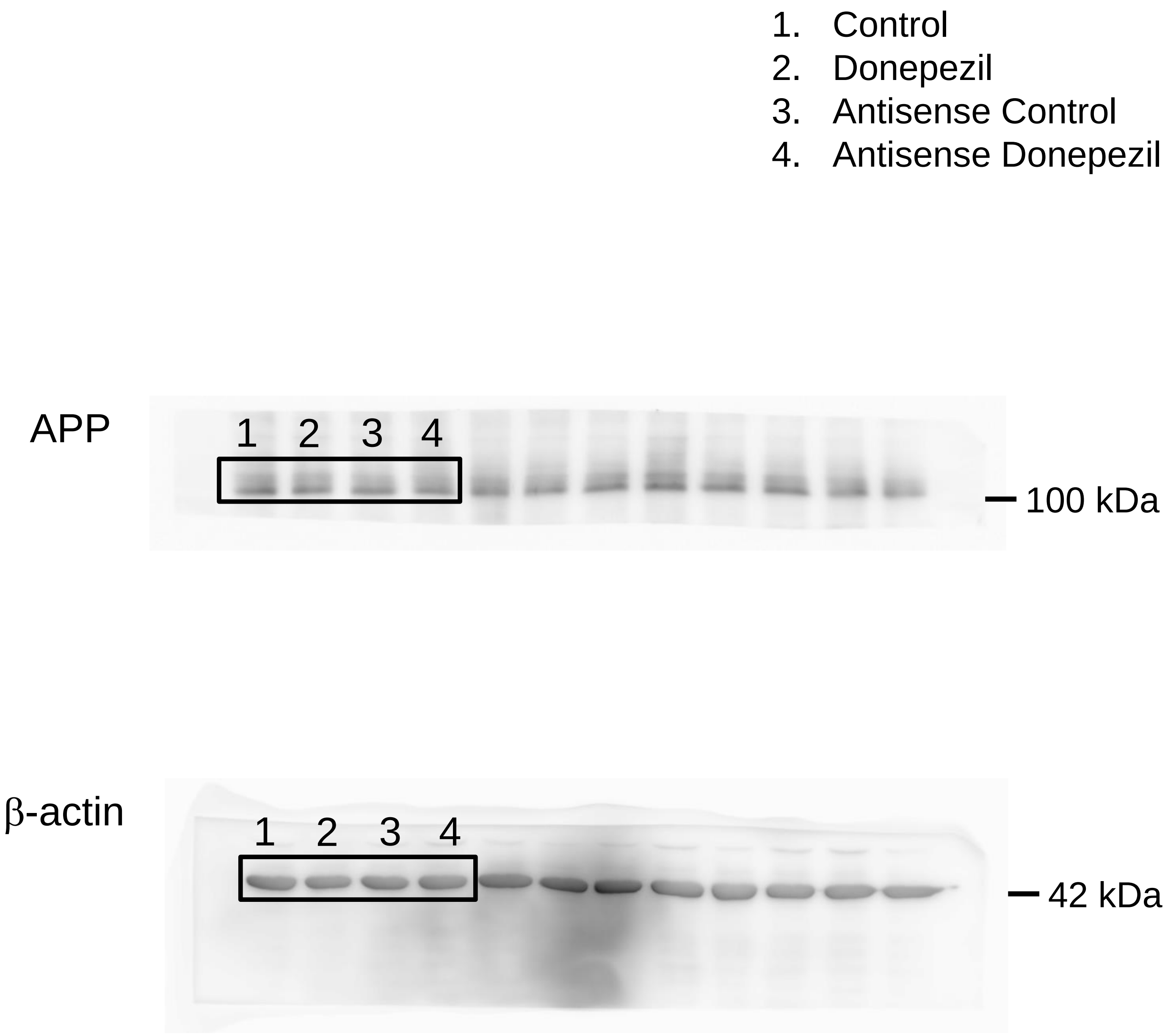

Supplemental figure S7. Original full length of blots for Fig. 7C. Boxes indicate areas shown in figure.

1. Control
2. Donepezil
3. Antisense Control
4. Antisense Donepezil

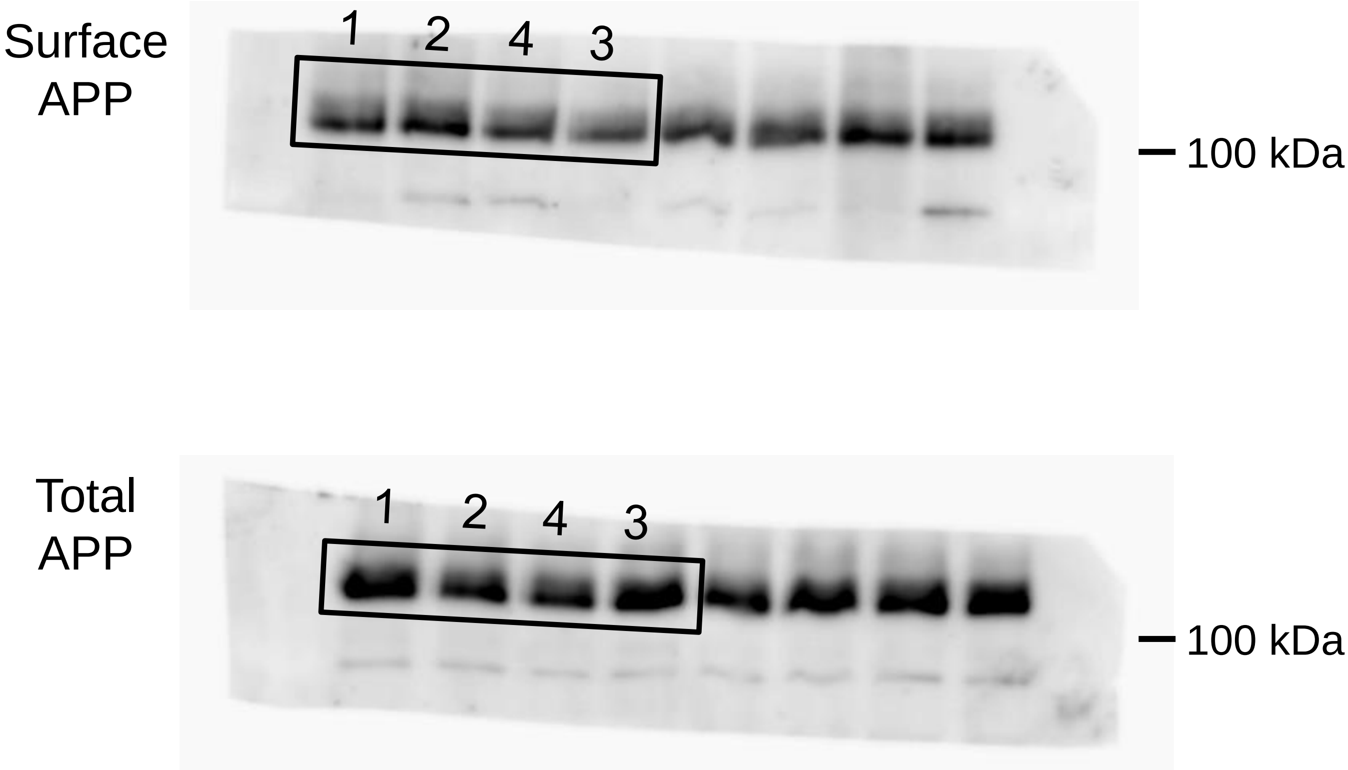

Supplemental figure S8. Original full length of blots for Fig. 8A. Boxes indicate areas shown in figure.
